# Factors Associated with Acute Kidney Injury Occurrence and Prognosis in Rhabdomyolysis at the Emergency Department

**DOI:** 10.3390/medicina60010105

**Published:** 2024-01-05

**Authors:** Jun Seok Seo, Inhwan Yeo, Changho Kim, Daeun Kim, Jeong-Hoon Lim, Kyoungtae Park, Jiwoo Jeong, Hojin Kwon, Yuna Cho, Sungyeon Park

**Affiliations:** 1 Department of Emergency Medicine, Dongguk University Ilsan Hospital, Dongguk University College of Medicine, Goyang-si 10326, Republic of Korea; drsjs75@dongguk.edu; 2 Department of Emergency Medicine, School of Medicine, Kyungpook National University, Daegu 41566, Republic of Korea; inani1113@gmail.com (I.Y.); da_eun22@naver.com (D.K.); 3 Department of Internal Medicine, School of Medicine, Kyungpook National University, Daegu 41566, Republic of Korea; 4 School of Medicine, Kyungpook National University, Daegu 41566, Republic of Korea; coxhdl80@naver.com (K.P.); ys06018@naver.com (J.J.); ghwls12589@naver.com (H.K.); yuna704@naver.com (Y.C.); jenne9926@gmail.com (S.P.)

**Keywords:** rhabdomyolysis, CPK, acute kidney injury, hemodialysis, mortality

## Abstract

*Background and Objectives:* This study aimed to analyze patients with rhabdomyolysis who presented to emergency departments and identify their distribution of related disease and prognostic factors. *Materials and Methods:* A retrospective cohort study was conducted on patients with rhabdomyolysis who presented to emergency departments over a 10-year period. Patient data, including patients’ demographic variables (sex and age), mode of arrival, final diagnosis, statin use, rhabdomyolysis trigger factors, and levels of serum creatine phosphokinase (CPK), myoglobin, creatinine, sodium, potassium, phosphate, calcium, and lactate, were analyzed. Univariate and multivariate logistic regression analyses were conducted to identify the predictive factors of acute kidney injury (AKI). *Results:* Among the patients, 268 (65.6%) were found to have trigger factors without underlying diseases. Furthermore, 115 (28.2%) patients developed AKI. This comprehensive study sheds light on the diverse factors influencing the occurrence of AKI in rhabdomyolysis and provides insights into AKI predictive markers. Furthermore, we analyzed the cases by dividing them into six groups: occurrence of AKI, occurrence of infection, and simple or complex rhabdomyolysis. CPK time course was found to be important in clinical prognosis, such as AKI occurrence, dialysis or not, and mortality. *Conclusions:* Age, statin use, elevated creatinine and lactate levels, and initial serum CPK level emerged as significant predictors of AKI. CPK time course was also found to be an important factor in predicting the clinical outcomes of patients with rhabdomyolysis.

## 1. Introduction

Rhabdomyolysis is a clinical syndrome that comprises the destruction of skeletal muscle with outflow of intracellular muscle content into the bloodstream [1] and a complex condition characterized by the breakdown of damaged muscle tissues, resulting in the release of intracellular muscle contents, including myoglobin, creatine phosphokinase (CPK), lactate dehydrogenase, and electrolytes [2,3]. This multifaceted syndrome is mainly caused by muscle injury, causing the release of various substances, including potassium, calcium, sodium, phosphate, myoglobin, and CPK [3,4].

Rhabdomyolysis has a diverse etiology, encompassing traumatic and nontraumatic factors, including drug and alcohol use, ischemia, infectious diseases, and myopathies [5,6,7]. Furthermore, rhabdomyolysis is known to be a potential complication of statin therapy [8].

Typically, the symptoms of rhabdomyolysis include muscle fatigue, pain, cramps, weakness, and, occasionally, an increase in muscle size. Myoglobinuria, described as the presence of myoglobin in the urine, serves as a significant diagnostic indicator of this condition. Laboratory tests consistently show a significant increase in serum CPK levels, frequently exceeding the normal range by five-fold, along with elevated urine myoglobin levels [9]. Severe complications caused by rhabdomyolysis include electrolyte imbalance, acute kidney injury (AKI), shock, and disseminated intravascular coagulation, which can be life-threatening and increase mortality rates by up to 37% among severe cases [2,10].

Among these complications, rhabdomyolysis-associated AKI is a critical systemic concern, accounting for 7–10% of all cases of acute renal failure [11]. In a retrospective study involving 2371 patients with rhabdomyolysis and CPK levels exceeding 5000 U/L, an alarming AKI incidence of 47.7% was observed, with a hospitalization-related mortality rate of 14.1% [2,12].

Nonetheless, the factors underlying AKI in rhabdomyolysis remain poorly understood, sparking ongoing debates [12]. The intricate interplay of factors contributing to this outcome warrants a more meticulous investigation of the pathophysiology of rhabdomyolysis-induced AKI. While myoglobin-induced renal toxicity is widely acknowledged, it is important to recognize that attributing AKI solely to myoglobin may oversimplify the multifaceted nature of this condition [10]. Forced alkaline diuresis helps alleviate the nephrotoxicity of myoglobin. Critically ill patients with acute renal failure are also at risk of developing multiple organ dysfunction syndrome, which can increase mortality rates [9]. To enhance the efficacy of preventive and therapeutic strategies for rhabdomyolysis-related kidney injury, a comprehensive understanding of the underlying pathophysiology is needed [8].

Therefore, this study aimed to identify the predictive factors of AKI occurrence in rhabdomyolysis, considering the variables including myoglobin, CPK, electrolyte levels, and CPK half-life. Furthermore, studies on the clinical spectrum of rhabdomyolysis in Korea have largely centered on case series and patient reports, with recent investigations mainly focusing on pediatric cases [10]. Studies investigating the association between AKI and rhabdomyolysis have been limited to trauma patients [11,12]. In addition, population-based studies involving non-trauma patients and adults have remained scarce since the 1994 paper by Choi SO [13]. Consequently, this study aimed to shed light on the clinical spectrum of patients with rhabdomyolysis who presented to the emergency department. It also aimed to identify the prognostic factors of AKI progression in patients diagnosed with rhabdomyolysis and analyze other factors to facilitate the clinical treatment of patients.

## 2. Material and Method

### 2.1. Patient Samples and Data Collection

**Figure 1:**
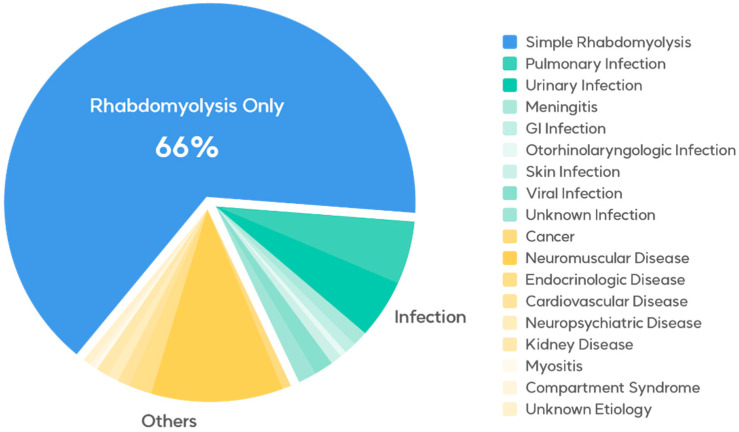
Medical conditions concurrent with rhabdomyolysis in the emergency department.

This retrospective cohort study included patients diagnosed with rhabdomyolysis (M6289) who presented to the emergency departments of Kyungpook National University Hospital and Kyungpook National University Chilgok Hospital from 1 January 2012 to 31 December 2021. To be eligible for the study, patients were required to exhibit serum CPK levels exceeding 950 U/L. Patients with elevated CPK levels due to dermatomyositis were excluded from the study. The patients’ data, including demographic variables (sex and age), mode of arrival, final diagnosis, statin use, rhabdomyolysis trigger factors, and levels of serum CPK, myoglobin, creatinine, sodium, potassium, phosphate, calcium, and lactate, were collected from their medical records and then thoroughly analyzed. The final diagnoses of the entire cohort are presented in Table 1 and Figure 1.

### 2.2. Definition

AKI was defined according to the 2012 Kidney Disease: Improving Global Outcomes (KDIGO) guidelines as follows: an increase in serum creatinine level of 0.3 mg/dL or more within 48 h, an increase in serum creatinine level of 1.5 times or more within 7 days, or urine output < 0.5 mL/kg/h within 6 h. The patients were divided into the “simple rhabdomyolysis” and “complex rhabdomyolysis” groups according to the conclusive diagnosis documented in the medical records. The former consisted of patients without co-occurring conditions, such as seizure and infection, whereas the latter consisted of patients with underlying conditions (Supplementary Table S1). Furthermore, patients with pulmonary or urinary infections, characterized by the presence of disease-causing bacteria or viruses in the body, were allocated to the “rhabdomyolysis with infection” group. The remaining patients were allocated to the “rhabdomyolysis without infection” group.

### 2.3. Statistical Analysis

Categorical variables were expressed as means with percentiles and compared using the chi-squared or Fisher’s exact test. Continuous variables were expressed as means with standard deviations (SDs) or medians with interquartile ranges (IQRs) and compared using Student’s *t*-test or the Mann–Whitney U test. Age and the cutoff levels of creatinine and lactate were determined on the basis of the largest Youden index rounded up to the nearest first decimal place. Furthermore, the initial cutoff levels of CPK, peak CPK, potassium, and phosphate were determined on the basis of the IQR. Subsequently, variables exhibiting significance in the univariate analysis were incorporated into multivariate models. Multivariate logistic regression was employed to compute adjusted odds ratios (ORs). Model discrimination was performed using the Hosmer–Lemeshow goodness-of-fit test. A two-sided *p*-value < 0.05 was considered to indicate statistical significance. All statistical analyses were conducted using the Statistical Package for Social Sciences version 27 (IBM, Armonk, NY, USA).

## 3. Results

### 3.1. Patients’ Characteristics

The characteristics of the 408 patients are presented in Table 1 and Table 2. The predominant underlying conditions associated with rhabdomyolysis were attributed to exercise, therapeutic drug use, alcohol consumption, and other factors, in the absence of concurrent illnesses, comprising 268 cases (65.6%). There were 66 (16.2%) infection-induced cases and 74 (18.2%) cases caused by other conditions.

Among the patients, 65.2% were men, with a mean age of 55 (SD, 29–73) years. A total of 206 patients (50.5%) arrived via a stretcher, the majority (95.6%) of whom were diagnosed with fasciitis for the first time. Statin use was observed in 22 (5.4%) patients. Furthermore, exercise emerged as the most common triggering factor for the condition in 113 (27.7%) patients, followed by infection in 66 (16.2%), illicit drug or alcohol use in 58 (14.2%), medical drug use in 37 (9.1%), seizure in 34 (8.3%), trauma and immobility in 35 (8.6%), climate change in 19 (4.7%), and other factors in 46 (11.3%). Laboratory characteristics are presented in Table 2.

### 3.2. Clinical Features and Outcomes Associated with AKI

Among the patients with rhabdomyolysis, 28.2% developed AKI, of whom 7.6% required renal replacement therapy (RRT) and 6.6% died during hospitalization. A comparison of the characteristics of these patients was performed; then, the patients were categorized into the AKI and non-AKI groups. The findings are presented in Table 1. The patients in the AKI group had a mean age of 64 (SD, 45.3–82.7) years, which was significantly higher than that of the patients in the non-AKI group, which was 47.7 (SD, 24.7–70.7) years (*p* < 0.001). The AKI group was more likely to arrive at the emergency department via a stretcher (*p* < 0.001), use statins (*p* = 0.019), and exhibit statistically significant differences from the non-AKI group in terms of trigger factors (*p* = 0.009). The initial CPK and peak CPK levels were 4441 (IQR, 1871–9055) U/L and 5705 (IQR, 2274–13,395) U/L, respectively, showing notable disparities compared with those in the non-AKI group (8036 (IQR, 2414–29,280) U/L and 10,015 (IQR, 3323–36,150) U/L, respectively; *p* < 0.001). In addition, the creatinine level of the AKI group was 1.9 (IQR, 1.4–3.4) mg/dL, which was significantly different from that of the non-AKI group (0.9 (IQR, 0.7–1.1) mg/dL; *p* < 0.001). Significant differences were also observed in the levels of potassium (*p* < 0.001), phosphate (*p* < 0.001), and lactate (*p* < 0.001) between the group.

### 3.3. Predictive Factors of AKI

In the AKI group, both univariate and multivariate logistic regression analyses were conducted to investigate the different factors influencing AKI development (Table 2). In the univariate analysis, age > 50.5 years (*p* < 0.001), mode of arrival (*p* < 0.001), statin use (*p* = 0.024), trigger factor (*p* = 0.02), presence of multiple trigger factors (*p* = 0.02), initial CPK level (*p* < 0.001), peak CPK level (*p* = 0.004), creatinine > 1.3 mg/dL (*p* < 0.001), potassium level (*p* < 0.001), phosphate level (*p* < 0.001), and lactate > 2.25 mmol/L (*p* < 0.001) were found to be associated with AKI.

**Figure 2:**
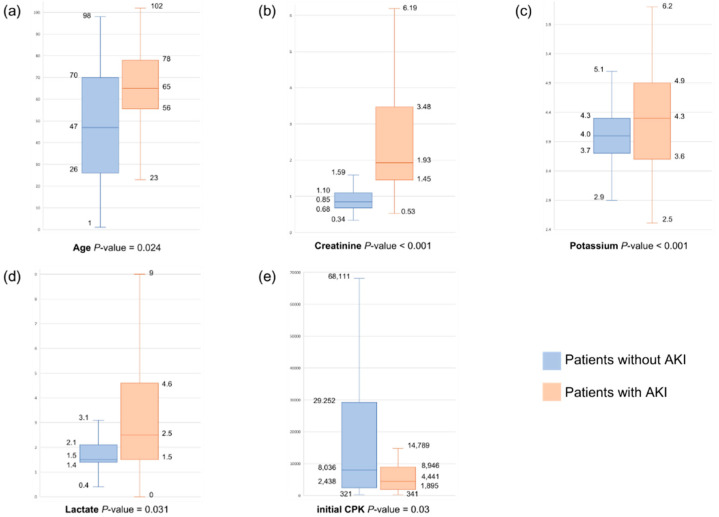
(**a**–**e**) Boxplots of significant variables between patients with and without AKI.

Multivariate analysis further revealed that age > 50.5 years (*p* = 0.024, OR = 3.01, 95% confidence interval (CI), 1.15–7.83), statin use (*p* = 0.02, OR = 5.14, 95% CI, 1.29–20.51), creatinine level > 1.3 mg/dL (*p* < 0.001, OR = 22.61, 95% CI, 10.30–49.60), and lactate level > 2.25 mmol/L (*p* = 0.031, OR = 2.35, 95% CI, 1.08–5.10) were independent factors significantly associated with AKI (Figure 2). The receiver operating characteristic curve of the significant variables is presented in Supplementary Table S1 and Supplementary Figure S1.

Regarding the initial CPK levels, compared with the reference group (Q1), the ORs for AKI occurrence were 5.96-fold higher in the initial CPK 1871~4441 U/L group (Q2) (95% CI, 1.42–24.99, *p* = 0.015) and 6.43-fold higher in Q3 (95% CI, 1.23–33.65, *p* = 0.028). However, Q4 exhibited a *p*-value of 0.34, and because the 95% CI included 1, OR 1 lacked statistical significance.

Regarding potassium levels, compared with Q1, the ORs for AKI occurrence decreased by 69% in Q2 (95% CI, 0.13–0.74, *p* = 0.008). Q3 and Q4 had *p*-values > 0.05, with 95% CIs encompassing 1, indicating no statistical significance.

### 3.4. Grouping Comparison

The patients were divided into six groups: patients without AKI vs. patients with AKI, simple rhabdomyolysis vs. complex rhabdomyolysis, and patients without infection vs. patients with infection. When the application of hemodialysis and mortality within these groups was analyzed, significant differences were observed.

**Figure 3:**
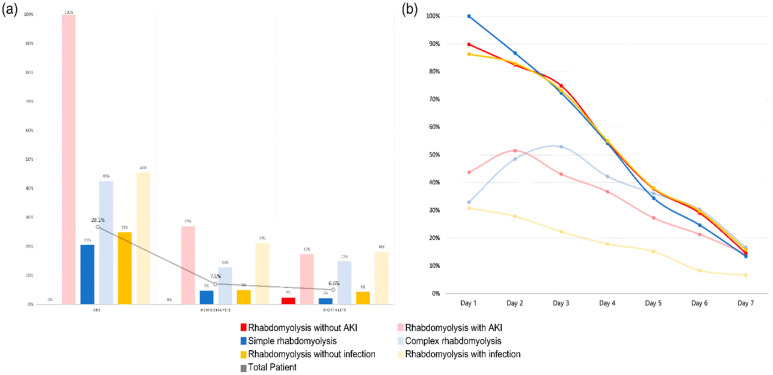
(**a**) AKI, HD, and mortality for each group. (**b**) CPK time course for each group.

As can be seen from Figure 3, the average CPK time course was over 1 week for each group, showing significant differences. Patients without AKI, simple rhabdomyolysis, and patients without infection, who had lower mortality rates, showed higher initial CPK levels, which subsequently declined rapidly over time compared with the other comparison groups. This suggests that in real emergency room clinical practice, the initial CPK levels may not be crucial when assessing the prognosis of patients with rhabdomyolysis. Furthermore, the CPK time course of over 1 week provides insights into the prediction of patient outcomes.

The characteristics of the simple and complex rhabdomyolysis groups are compared and summarized in Table 3. The proportion of men was similar between the simple and complex rhabdomyolysis groups (64.8% and 66.0%, respectively) and these groups exhibited distinct mean ages, with values of 47.9 (SD, 24.9–70.9) and 60.7 (SD, 40.0–81.4) years for the former and latter groups, respectively. Notably, significant differences were observed in terms of statin use (*p* = 0.043), trigger factor (*p* < 0.001), and initial levels of CPK (*p* < 0.001), peak CPK (*p* < 0.001), creatinine (*p* < 0.001), calcium (*p* < 0.001), and lactate (*p* < 0.001).

Among the patients in the complex rhabdomyolysis group, 42.6% developed AKI, of whom 12.8% required RRT and 14.9% died during hospitalization (Table 3). Table 3 presents a comparative analysis between the groups with and without infection occurrences in the entire cohort. The average CPK level changes for each group are illustrated in Figure 3, whereas AKI occurrence, requirement of hemodialysis, and mortality rate are illustrated in Figure 3.

### 3.5. CPK Time Course

The serum CPK time course across all the groups is presented in Figure 3. The daily average serum CPK levels were measured for each group and visualized as time course curves. The 100% interval on the graph indicates the initial mean CPK level of the “simple rhabdomyolysis” group, which had the highest initial CPK mean level among all the six groups. Specifically, the mean initial CPK level for the “rhabdomyolysis without AKI” group was 26,866 (SD ± 2363) U/L, showing a significant difference from that in the “rhabdomyolysis with AKI” group, which had a mean of 13,074 (SD ± 2667) U/L (*p* < 0.001). Also, the mean initial CPK level for the “simple rhabdomyolysis” group was 29,980 (SD ± 2694) U/L, showing a significant difference from that in the “complex rhabdomyolysis” group, which had a mean of 9856 (SD ± 1316) U/L (*p* < 0.001). The “patient without infection” and “patient with infection” groups exhibited distinct initial CPK, with values of 25,814 (SD ± 2194) U/L and 8282 (SD ± 1397) U/L, respectively (*p* < 0.001).

Furthermore, when “rhabdomyolysis with AKI” vs. “rhabdomyolysis without AKI”, “simple” vs. “complex” rhabdomyolysis, and “rhabdomyolysis without” vs. “with infection” were compared, no significant differences were observed in the mean serum CPK levels measured on the seventh day (Supplementary Table S2).

However, there was a significant difference in mortality among the groups (Table 3).

## 4. Discussion

Rhabdomyolysis is a complex condition that is characterized by the breakdown of damaged muscle tissues, resulting in the release of intracellular muscle contents. In this study, we first characterized the disease spectrum of patients with rhabdomyolysis who presented to the emergency departments of the aforementioned hospitals. Second, we identified the risk factors for AKI. Lastly, by discerning the pattern of CPK levels over time, we anticipated the likelihood of AKI occurrence, need for hemodialysis, and mortality rates.

Until recently, the risk of AKI or death was mainly estimated using the maximum peak of serum CPK, with a suggested discriminative cutoff value of 5000 U/L. However, recent retrospective studies have demonstrated the low predictive value of the maximum peak of CPK as an isolated predictive biomarker and alternatively proposed the integration of this parameter within a composite model [14]. Also, in a previous study of spinning exercise-related rhabdomyolysis, the patients exhibited CPK levels exceeding 11,000 U/L within a 9-month period, yet none developed AKI [15].

In this study, one of the most interesting findings was the unexpected observation of higher initial CPK levels in the non-AKI group than in the AKI group. This novel result challenges the conventional understanding that elevated CPK levels are directly associated with increased risk for AKI in patients with rhabdomyolysis. Our study sheds light on the intricate interplay of various factors influencing AKI occurrence in this context. Therefore, as previously stated, the statement that in-hospital mortality is high in cases of simple rhabdomyolysis or rhabdomyolysis without AKI is not necessarily true, even when the initial CPK levels are elevated.

Age was found to be a significant predictor of AKI occurrence, with patients aged above 50.5 years exhibiting a higher risk (OR = 3.01, 95% CI, 1.15–7.83, *p* = 0.024). This finding is consistent with those of previous studies [16,17] and highlights the significance of age as a contributing factor to AKI development in patients with rhabdomyolysis. Similarly, statin use was associated with increased risk for AKI development (OR = 5.14, 95% CI, 1.29–20.51, *p* = 0.02), emphasizing the potential myotoxic effects of statins [18]. Elevated creatinine level emerged as a robust predictor of AKI in patients with rhabdomyolysis (OR = 22.61, 95% CI, 10.30–49.60, *p* < 0.001), which is consistent with the established evidence of its significance in the assessment of renal impairment [19]. Furthermore, an elevated lactate level was found to be an independent predictor of AKI (OR = 2.35, 95% CI, 1.08–5.10, *p* = 0.031), suggesting that metabolic disturbances play a role in renal compromise among patients with rhabdomyolysis [20].

Interestingly, our study challenges the traditional notion that potassium plays a role in the development of AKI. Higher potassium levels were found to be inversely associated with AKI development risk, with the OR for AKI occurrence decreasing by 69% in Q2 (95% CI, 0.13–0.74, *p* = 0.008). However, this association did not hold significance in Q3 and Q4, suggesting a potential threshold effect.

These findings collectively underscore the complex nature of rhabdomyolysis and its diverse clinical presentations. Comparison between simple and complex cases supports the heterogeneous nature of this condition, highlighting the need for individualized risk assessment and tailored management strategies to prevent AKI and optimize patient outcomes.

## 5. Conclusions

In conclusion, our study’s novel finding of higher initial CPK levels in the non-AKI group challenges the traditional understanding that CPK is directly associated with AKI occurrence. Our comprehensive analysis revealed that age, statin use, elevated creatinine and lactate levels, and initial CPK levels were significant predictors of AKI development. It provides valuable insights into the intricate interplay of factors influencing AKI development in patients with rhabdomyolysis. To improve risk assessment strategies and optimize patient care in the context of this complex condition, further research is warranted.

## 6. Key Message

We conducted a retrospective analysis of patients with rhabdomyolysis who presented to emergency departments over a 10-year period. Among the cases, 65.6% involved no underlying diseases, 16.2% were associated with infection, and 18.2% involved other causes. To predict the clinical prognosis of these patients, we investigated the predictive factors of acute kidney injury (AKI) and explored the clinical spectrum of the disease as well as the outcomes in the emergency department. The significant predictors of AKI included age, statin use, elevated creatinine and lactate levels, and elevated initial creatine phosphokinase (CPK) levels. CPK time course also emerged as a crucial factor for predicting the clinical outcomes of patients with rhabdomyolysis.

## 7. Limitations

Previous studies have demonstrated that serum CPK levels begin to increase approximately 2–12 h after the onset of muscle injury, peak within 24–72 h, and then decline at a relatively constant rate of approximately 40% of the previous day’s value. It is CPK that is commonly measured; however, it is not directly implicated in the pathogenesis of rhabdomyolysis, rather it is myoglobin [21]. Myoglobin is the pathogenic factor in rhabdomyolysis-induced AKI, but it is rarely directly measured in serum and urine. The levels of serum myoglobin peak well before CPK levels and then decline quickly due to rapid and unpredictable metabolism. Therefore, myoglobin exhibits low sensitivity for the diagnosis of rhabdomyolysis. In addition, rhabdomyolysis-induced AKI is often associated with a more rapid increase in plasma creatinine levels than other types of AKI [22]. Thus, in future studies, we can provide a scholarly examination of the clinical prediction and prognosis of AKI by conducting a prospective study that includes not only the CPK time course but also data on plasma creatinine time course, serum myoglobin, urine myoglobin, CPK half-life, and other relevant tests over time.

## Figures and Tables

**Table 1:** Clinical and laboratory characteristics of patients with rhabdomyolysis.

Characteristic	All Patients (*n* = 408)	Patients without AKI (*n* = 293)	Patients with AKI (*n* = 115)	*p*-Value
Sex, male	266 (65.2)	196 (66.9)	70 (60.9)	0.250
Age, years	55.0 (29.0–73.0)	47.7 (24.7–70.7)	64.0 (45.3–82.7)	<0.001
Mode of arrival				<0.001
Stretcher	206 (50.5)	127 (43.3)	75 (65.2)	
Walk	202 (49.5)	166 (56.7)	40 (34.8)	
Diagnosis status				0.565
Initial diagnosis	390 (95.6)	279 (95.2)	111 (96.5)	
Recurrent	18 (4.4)	14 (4.8)	4 (3.5)	
Statin use	22 (5.4)	11 (3.8)	11 (9.6)	0.019
Triggering factor				0.009
Exercise	113 (27.7)	74 (25.3)	39 (33.9)	
Infection	66 (16.2)	45 (15.4)	21 (18.3)	
Illicit drugs, alcohol	58 (14.2)	45 (15.4)	13 (11.3)	
Medical drugs	37 (9.1)	34 (11.6)	3 (2.6)	
Seizure	34 (8.3)	28 (9.6)	6 (5.2)	
Trauma, immobility	35 (8.6)	26 (8.9)	9 (7.8)	
Climate change	19 (4.7)	9 (3.1)	10 (8.7)	
Others	46 (11.3)	32 (10.9)	14 (12.2)	
Multiple triggering factors	104 (25.5)	66 (22.5)	38 (33.0)	0.032
Initial CPK, U/L	7800 (2143–20,000)	8036 (2414–29,280)	4441 (1871–9055)	<0.001
Peak CPK, U/L	7859 (3003–26,369)	10,015 (3323–36,150)	5705 (2274–13,395)	<0.001
CPK half-life, day	1.5 (1.0–2.0)	1.5 (1.4–2.0)	1.5 (1.0–2.0)	0.554
Myoglobin, ng/mL	2888 (1000–10,762)	2347 (902–10,000)	4119 (1200–12,000)	0.061
Creatinine, mg/dL	1.02 (0.74–1.58)	0.9 (0.7–1.1)	1.9 (1.4–3.4)	<0.001
Sodium, mmol/L	138.0 (136.0–141.0)	138.0 (136.0–141.0)	138.0 (135.0–141.0)	0.734
Potassium, mmol/L	4.0 (3.7–4.4)	4.0 (3.7–4.3)	4.3 (3.6–4.9)	<0.001
Phosphate, mmol/L	3.5 (2.9–4.3)	3.5 (2.9–4.0)	4.2 (3.0–5.4)	<0.001
Calcium, mmol/L	9.0 (8.2–9.3)	9.0 (8.3–9.3)	8.8 (8.0–9.3)	0.135
Lactate, mmol/L	1.5 (1.4–2.7)	1.5 (1.4–2.1)	2.5 (1.5–4.7)	<0.001
Outcomes				
Acute kidney injury	115 (28.2)	-	-	-
Hemodialysis	31 (7.6)	0 (0)	31 (27.0)	<0.0001
Mortality	27 (6.6)	7 (2.4)	20 (17.4)	<0.001

**Table 2:** Clinical and laboratory factors contributing to rhabdomyolysis in patients with AKI using logistic regression.

Variables	Univariate Model			Multivariate Model		
	Crude OR	95% CI	*p*-Value	Adjusted OR	95% CI	*p*-Value
Age, years > 50.5	5.87	3.44–10.02	<0.001	3.01	1.15–7.83	0.024
Mode of arrival			<0.001			0.537
Stretcher	1.00			1.00		
Walk	2.45	1.57–3.84		0.78	0.35–1.73	
Statin use	2.71	1.14–6.44	0.024	5.14	1.29–20.51	0.020
Triggering factor			0.020			0.131
Exercise	1.13	0.59–2.16	0.124	0.47	0.13–1.72	0.251
Infection	1.00	-		1.00	-	
Illicit drugs, alcohol	0.62	0.28–1.39	0.004	0.42	0.13–1.41	0.161
Medical drugs	0.19	0.05–0.69	0.120	0.21	0.05–0.84	0.028
Seizure	0.46	0.17–1.28	<0.001	0.89	0.24–3.29	0.857
Trauma, immobility	0.74	0.30–1.86	0.280	0.52	0.11–2.33	0.084
Climate change	2.38	0.84–6.73	0.506	1.56	0.48–5.01	0.39
Others	0.94	0.42–2.12	0.804	1.07	0.49–2.32	0.459
Multiple triggering factors	1.70	1.06–2.73	0.029	1.07	0.49–2.32	0.861
Initial CPK			<0.001			0.030
~1871 U/L	Reference (1.00)			Reference (1.00)		
1871~4441 U/L	1.36	0.71–2.58	0.354	5.96	1.42–24.99	0.015
4441~9055 U/L	1.30	0.68–2.46	0.425	6.43	1.23–33.65	0.028
9055 U/L~	0.42	0.23–0.77	0.005	2.39	0.39–14.59	0.343
Peak CPK			0.004			0.521
−2274 U/L	Reference (1.00)			Reference (1.00)		
2274~5705 U/L	1.12	0.59–2.14	0.725	0.55	0.14–2.26	0.410
5705~13,395 U/L	0.92	0.49–1.72	0.787	0.30	0.06–1.64	0.166
13,395 U/L~	0.41	0.22–0.76	0.004	0.48	0.07–3.16	0.446
Chronic kidney disease	34.89	18.66–65.25	<0.001	22.61	10.30–49.60	<0.001
Potassium			<0.001			<0.001
~3.6 mmol/L	Reference (1.00)			Reference (1.00)		
3.6~4.3 mmol/L	0.37	0.21–0.65	<0.001	0.31	0.13–0.74	0.008
4.3~4.9 mmol/L	1.13	0.60–2.13	0.711	0.62	0.23–1.66	0.341
4.9 mmol/L~	8.50	3.36–21.52	<0.001	3.85	0.99–14.92	0.051
Phosphate			<0.001			0.709
~3.0 mmol/L	Reference (1.00)			Reference (1.00)		
3.0~4.2 mmol/L	0.70	0.40–1.22	0.209	1.54	0.66–3.61	0.321
4.2~5.4 mmol/L	1.76	0.93–3.36	0.085	1.20	0.44–3.31	0.724
5.4 mmol/L~	7.24	3.27–16.04	<0.001	1.71	0.54–5.42	0.361
Lactate > 2.25 mmol/L	5.31	3.33–8.46	<0.001	2.35	1.08–5.10	0.031

**Table 3:** Clinical and laboratory characteristics of patients within all groups.

Characteristics	Patients with Simple Rhabdomyolysis (*n* = 267)	Patients with Complex Rhabdomyolysis (*n* = 141)	*p*-Value	Patients without Infection (*n* = 342)	Patients with Infection (*n* = 66)	*p*-Value
Sex, male	173 (64.8)	93 (66.0)	0.815	221 (64.6)	45 (68.2)	0.578
Age, years	47.9 (24.9–70.9)	60.7 (40.0–81.4)	<0.001	51.5 (27.8–71.0)	68.5 (52.8–78.0)	<0.001
Mode of arrival			<0.001			0.025
Stretcher	162 (60.7)	44 (31.2)		161 (47.1)	41 (62.1)	
Walk	105 (39.3)	97 (68.8)		181 (52.9)	25 (37.9)	
Diagnosis status			0.911			0.329
Initial diagnosis	255 (95.5)	135 (95.7)		325 (95.0)	65 (98.5)	
Recurrent	12 (4.5)	6 (4.3)		17 (5.0)	1 (1.5)	
Statin use	10 (3.7)	12 (8.5)	0.043	18 (5.3)	4 (6.1)	0.767
Triggering factor			<0.001			<0.001
Exercise	113 (42.3)	0 (0)		113 (33.0)	0 (0)	
Infection	0 (0)	66 (46.8)		0 (0)	66 (100)	
Illicit drugs, alcohol	58 (21.7)	0 (0)		58 (17.0)	0 (0)	
Medical drugs	36 (13.5)	1 (0.7)		37 (10.8)	0 (0)	
Seizure	0 (0)	34 (24.1)		34 (9.9)	0 (0)	
Trauma, immobility	35 (13.1)	0 (0)		35 (10.2)	0 (0)	
Climate change	19 (7.1)	0 (0)		19 (5.6)	0 (0)	
Others	6 (2.2)	40 (28.4)		46 (13.5)	0 (0)	
Multiple triggering factors	68 (25.5)	36 (25.5)	0.989	87 (25.4)	17 (25.8)	0.957
Initial CPK, U/L	8830 (3073–31,964)	4490 (1697–9692)	<0.001	7859 (2448–23,778)	4121 (1729–9017)	<0.001
Peak CPK, U/L	10,286 (3667–43,038)	5837 (1853–14,578)	<0.001	8945 (3276–31,639)	5298 (1831–10,777)	<0.001
CPK half-life, day	1.5 (1.0–2.0)	1.5 (1.5–2.0)	0.597	1.5 (1.0–2.0)	1.5 (1.4–2.0)	0.556
Myoglobin, ng/mL	3044 (1000–11,324)	2862 (1013–9814)	0.342	2921 (1000–10,798)	2747 (1083–10,131)	0.919
Creatinine, mg/dL	0.92 (0.70–1.37)	1.26 (0.87–2.34)	<0.001	0.99 (0.72–1.49)	1.3 (0.91–2.13)	0.002
Sodium, mmol/L	137.7 (6.1)	137.6 (9.2)	0.901	138.0 (136.0–141.0)	138.0 (134.0–143.0)	0.043
Potassium, mmol/L	4.1 (0.7)	4.0 (0.7)	0.370	4.1 (3.7–4.4)	3.9 (3.5–4.4)	0.703
Phosphate, mmol/L	3.5 (3.0–4.3)	3.5 (2.9–4.3)	0.812	3.5 (3.0–4.3)	3.5 (2.7–4.3)	0.514
Calcium, mmol/L	9.1 (8.4–9.3)	8.7 (7.9–9.2)	<0.001	9.0 (8.2–9.3)	8.6 (7.7–9.2)	0.001
Lactate, mmol/L	1.5 (1.4–2.1)	2.2 (1.5–3.6)	<0.001	1.5 (1.4–2.4)	2.3 (1.5–3.6)	0.015
Acute kidney injury	55 (20.6)	60 (42.6)	<0.001	85 (24.9)	30 (45.5)	<0.001
Hemodialysis	13 (4.9)	18 (12.8)	0.004	17 (5.0)	14 (21.2)	<0.001
Mortality	6 (2.2)	21 (14.9)	<0.001	15 (4.4)	12 (18.2)	<0.001

## Data Availability

No new data were created or analyzed in this study.

## Supplementary Materials

The following supporting information can be downloaded at: https://www.mdpi.com/article/10.3390/medicina60010105/s1. Table S1: Grouping criteria according to the final diagnosis. Table S2: Clinical and laboratory characteristics of patients within all groups. Table S3: Sensitivity, specificity, and area under the curve (AUC) of the predictive model for AKI based on receiver operating characteristic curve analysis. Figure S1: ROC curve of significant variables in the multivariable logistic regression.
