# Supplementary material for: Factors Associated with Acute Kidney Injury Occurrence and Prognosis in Rhabdomyolysis at the Emergency Department
Source: Medicina (Kaunas). 2024 Jan 5;60(1):105. doi: 10.3390/medicina60010105 (PMC10819221; doi:10.3390/medicina60010105)
Supplement: Supplementary file 1 [file medicina-60-00105-s001.zip › medicina-2744242-supplementary.pdf]

Supplementary Information

Supplementary Table S1

Grouping criteria according to the final diagnosis

Simple rhabdomyolysis	Complex rhabdomyolysis
Drug-induced	Pulmonary infection
Exercise	Urinary infection
Burn	Meningitis
Heat stroke	Gastrointestinal infection
Hypothermia	Otorhinolaryngologic infection
Trauma	Skin infection
Bite	Viral infection
CO intoxication	Unknown infection
	Cancer
	Neuromuscular disease
	Endocrinological disease
	Cardiovascular disease
	Neuropsychiatric disease
	Kidney disease
	Myositis
	Compartment syndrome
	Unknown etiology

Supplementary Table S2

Clinical and laboratory characteristics of patients within all groups

Serum CPK, U/L	Patients without AKI (*n*= 293)	Patients with AKI (*n* = 115)	P-value	Patients with simple rhabdomyolysis (*n* = 267)	Patients with complex rhabdomyolysis (*n* = 141)	P-value	Patients without infection (*n* = 342)	Patients with infection (*n* = 66)	P-value
Initial CPK (Day 1)	26,866 (2,363)	13,074 (2,667)	<0.001	29,908 (2,694)	9,856 (1,316)	<0.001	25,814 (2,194)	8,282 (1,397)	<0.001
Day 2	24,644 (2,567)	15,401 (4,121)	0.001	25,921 (2,942)	14,499 (2,901)	0.002	24,803 (2,571)	7,479 (1,202)	<0.001
Day 3	22,412 (2,828)	12,879 (3,439)	0.007	21,633 (2,809)	15,826 (3,810)	0.120	21,898 (2,583)	5,999 (1,030)	0.002
Day 4	16,345 (2,327)	10,988 (3,137)	0.158	16,212 (2,293)	12,636 (3,464)	0.087	16,463 (2,160)	4,814 (977)	0.009
Day 5	11,308 (2,092)	8,160 (2,438)	0.485	10,301 (1,744)	10,797 (3,483)	0.836	11,376 (1,883)	4,079 (833)	0.066
Day 6	8,674 (2,563)	6,373 (2,336)	0.735	7,382 (1,589)	9,067 (4,489)	0.515	8,878 (2,216)	2,245 (424)	0.130
Day 7	4,366 (1,000)	4,265 (1,466)	0.879	4,020 (966)	4,991 (1,567)	0.289	4,723 (934)	1,788 (413)	0.579

Mean (±standard deviation)

Supplementary Table S3

Sensitivity, specificity, and area under the curve (AUC) of the predictive model for AKI based on receiver operating characteristic curve analysis

Variable	Sensitivity	Specificity	Youden index	AUC (95% CI)	P-value
Age, years	0.826	0.553	0.379	.703 (0.65–0.76)	<0.001
Creatinine	0.870	0.840	0.709	.887 (0.85–0.92)	<0.001
Potassium	0.417	0.911	0.329	.614 (0.54–0.68)	<0.001
Lactate	0.583	0.792	0.374	.684 (0.62–0.75)	<0.001
Initial CPK	0.913	0.102	0.015	.386 (0.33–0.44)	<0.001

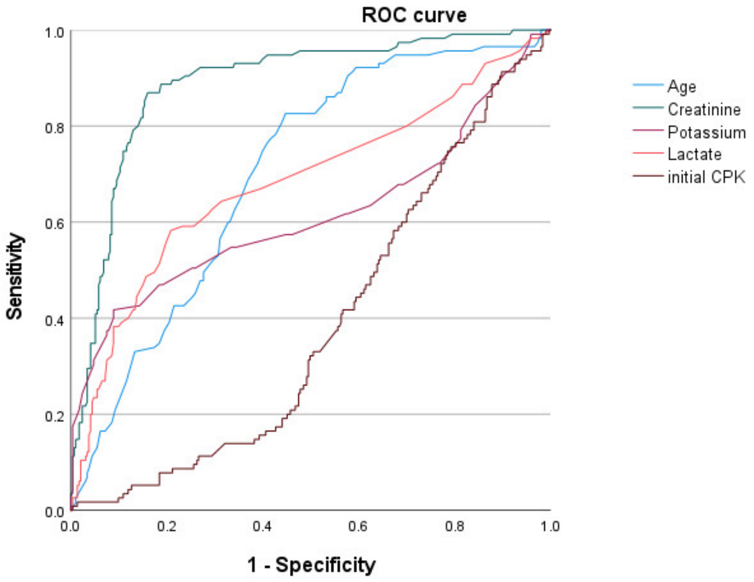

Supplementary Figure S1. ROC curve of significant variables in the multivariable logistic regression
